# Synthetic and Natural Inhibitors of Mortalin for Cancer Therapy

**DOI:** 10.3390/cancers16203470

**Published:** 2024-10-13

**Authors:** Shruti Kaushal, Samriddhi Gupta, Seyad Shefrin, Dhvani Sandip Vora, Sunil C. Kaul, Durai Sundar, Renu Wadhwa, Jaspreet Kaur Dhanjal

**Affiliations:** 1 Department of Computational Biology, Indraprastha Institute of Information Technology (IIIT) Delhi, Okhla Industrial Estate, Phase III, New Delhi 110020, India; shrutii@iiitd.ac.in (S.K.); samriddhig@iiitd.ac.in (S.G.); dhvani.vora@dbeb.iitd.ac.in (D.S.V.); 2 Department of Biochemical Engineering and Biotechnology, Indian Institute of Technology (IIT) Delhi, New Delhi 110016, India; bez188440@dbeb.iitd.ac.in (S.S.); sundar@dbeb.iitd.ac.in (D.S.); 3 AIST-INDIA DAILAB, National Institute of Advanced Industrial Science & Technology (AIST), Central 4-1, Tsukuba 305-8565, Japan; s-kaul@aist.go.jp; 4 Institute of Bioinformatics and Applied Biotechnology (IBAB), Bengaluru 560100, India

**Keywords:** mortalin, Hsp70, chaperone, cancer, natural and synthetic inhibitors

## Abstract

**Simple Summary:**

Mortalin is a heat shock protein 70 stress chaperone family member with variable subcellular localization in normal and cancer cells. It is an essential protein with multiple functions that support and promote proliferation, endorsed by its enriched expression in various cancers. Due to its prime involvement in stress adaptation and carcinogenesis, regulating Mortalin expression and function is a viable therapeutic avenue.

**Abstract:**

Upregulation of stress chaperone Mortalin has been closely linked to the malignant transformation of cells, tumorigenesis, the progression of tumors to highly aggressive stages, metastasis, drug resistance, and relapse. Various in vitro and in vivo assays have provided evidence of the critical role of Mortalin upregulation in promoting cancer cell characteristics, including proliferation, migration, invasion, and the inhibition of apoptosis, a consistent feature of most cancers. Given its critical role in several steps in oncogenesis and multi-modes of action, Mortalin presents a promising target for cancer therapy. Consequently, Mortalin inhibitors are emerging as potential anti-cancer drugs. In this review, we discuss various inhibitors of Mortalin (peptides, small RNAs, natural and synthetic compounds, and antibodies), elucidating their anti-cancer potentials.

## 1. Mortalin Structure and Function

The Hsp70 chaperone family is a primary component of stress response and homeostasis. These chaperones regulate the folding of newly translated peptides, thereby preventing anomalous accumulation. Family members of Hsp70 chaperone in eukaryotes are mainly found in the endoplasmic reticulum (BiP/Grp78), cytosol (Hsp70 and near homologs), and mitochondria [1,2,3] and play a key role in protein quality control by assisting in the correct folding and degradation of misfolded/dysfunctional proteins. Mortalin was also termed as mitochondrial stress-70 protein (mtHsp70), p66mot-1, HspA9/HspA9B, or Grp75, as discovered in various experimental approaches endorsing its multi-functionality early on. It has been detected in various subcellular niches, including cytoplasmic vesicles [4], endoplasmic reticulum [5], and cytosol [5,6,7], with predominant residence in the mitochondria [6,7,8]. Differential staining patterns of Mortalin in normal and immortal cells were reported, wherein the pancytoplasmic distribution was associated with the mortal phenotype, and the perinuclear distribution was associated with the immortal phenotype [7]. Note that the shift of the Mortalin staining pattern from perinuclear to pancytoplasmic has been associated with the induction of senescence in cancer cells. For example, cancer cells treated with Mortalin shRNA, withaferin-A, Withanone, Cucurbitacin-B, or fucoxanthin showed growth arrest. They displayed pancytoplasmic Mortalin staining in contrast to the perinuclear type in control untreated cells [7,9,10,11].

**Figure 1:**
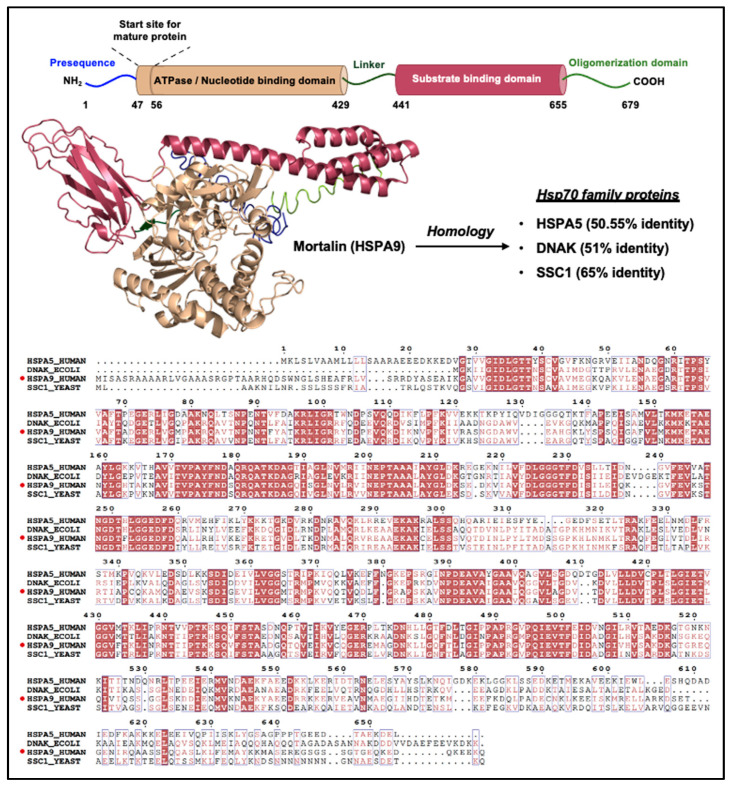
Schematic representation of Mortalin structure and homology. The domain organization (constructed using I-TASSER [12]) and sequence-based homology with other closely related members of the Hsp70 family of proteins are shown.

Encoded by the HSPA9B gene on Chromosome 5q31 (18 kb region), 2.8 kb Mortalin transcript codes for a ~70 kDa protein possessing 679 amino acid residues. It has an N-terminal 46-amino-acid-residue-long mitochondrial localization signal peptide, followed by a nucleotide-binding region (NBD; ~42-kDa: 47–429 amino acid residues) possessing an ATPase domain, an interdomain linker (430–440 amino acid residues), a substrate-binding domain (SBD; ~25-kDa: 441–655 amino acid residues), and an oligomerization domain (656–679 amino acid residues) at the C terminus, similar to other chaperones of the Hsp70 family [1]. The SBD consists of two parts: an α-helical domain forming a lid-like structure (SBDα) and a sandwich-like domain made up of β sheets (SBDβ), as shown in Figure 1. The latter binds to sequences rich in basic and hydrophobic amino acid residues. This peptide binding site of Hsp70 family members, in its high-substrate-affinity ADP-bound conformation, remains occupied with the five helices of SBDα. SBD and NBD do not interact in this configuration but are linked by a brief interdomain linker. With ADP-ATP exchange, the SBDα undergoes a substantial conformational shift, opening the peptide-binding site. The interaction of SBDβ and the interdomain linker to the NBD is coupled with a change in SBDβ conformation, thereby releasing the substrate protein. These processes are responsible for the allosteric regulation of the chaperones brought about by nucleotides [1,2,7].

The human Hsp70 subfamily consists of 13 proteins, including HspA1A, HspA1B, HspA1L, HspA2, HspA5, HspA6, HspA7, HspA8, HspA9, HspA12A, HspA12B, HspA13b, and HspA14 [7,13]. Mortalin has the highest sequence identity, 50.55%, with HspA5 (also known as Grp78, BiP, or MIF2). Mortalin also exhibits significant sequence identity with other members of the Hsp70 family, including 51% identity with evolutionary predecessor Escherichia coli DnaK and 65% identity with Saccharomyces cerevisiae SSC1p [7]. The sequence comparison among proteins is also shown in Figure 1. These conserved domains have been established to regulate mitochondrial function, dynamics, morphology, and homeostasis [14,15].

Subcellular localization studies have revealed mitochondria as a significant niche of Mortalin, while it is found in other cellular compartments, such as the nucleus, endoplasmic reticulum, cytoplasm, plasma membrane, and exosome [5,16]. Furthermore, it has been assigned niche-specific functions. For example, Mortalin coordinates with TIM and TOM membrane-associated proteins in mitochondria and plays a crucial role in the unfolding, translocation, and folding of imported proteins [17]. It was shown to play an essential role in the synthesis of iron–sulfur clusters, as well as their insertion into Fe-S apoproteins [18]. Mortalin was shown to enable the interaction of inositol 1, 4, 5-triphosphate receptor (IP3R), present on the surface of the endoplasmic reticulum, with the voltage-dependent anion channel (VDAC1) present on the surface of the mitochondrial membrane, thereby facilitating the movement of Ca^2+^ from the endoplasmic reticulum lumen to the mitochondrial matrix [19,20,21]. During redox stress, it mediates the mitochondrial unfolded protein response (UPR) as one of the chief chaperones responsible for properly folding the mitochondrial proteins [22,23]. Mortalin is involved in telomere maintenance mechanisms in the nucleus and is responsible for centrosome duplication during DNA replication [16]. It is also known to play a role in the release of exosomes. Mortalin, when present in exosomes, is responsible for cellular immunity and evading the immune system. The ATPase binding domain of Mortalin binds to the C5b-9 complex, thereby preventing it from forming pores in the cell and thus preventing cell lysis [24,25,26,27].

## 2. Role of Mortalin in Cancer

A large number of in silico and experimental studies have addressed the physiological function of Mortalin and its involvement in various pathological conditions, including hyperthyroidism, metabolic stress, thyroid hormone treatment, glucose deficiency, and environmental stresses, including ionizing radiation, cytotoxins, and calcium dysregulation [14,15,28]. Its dysregulation has also been linked to multiple neurological disorders, such as Alzheimer’s [29], Parkinson’s [30,31], Huntington [32], Amyotrophic Lateral Sclerosis (ALS) [33], EVEN-PLUS syndrome [34], and Congenital Sideroblastic Anemias (CSAs) [35]. The upregulation of Mortalin expression is often linked to protecting cells against stress and lethal circumstances [7,36,37]. At the same time, it is found consistently enriched in various cancer cells, wherein it plays vital roles in their uncontrolled proliferation, anti-apoptosis, and metastatic properties [38,39,40,41]. Since proliferative and metastatic pathways are interlinked by feed-forward mechanisms to drug resistance and tumor recurrence [42,43], Mortalin has been proposed as an important therapeutic target.

**Figure 2:**
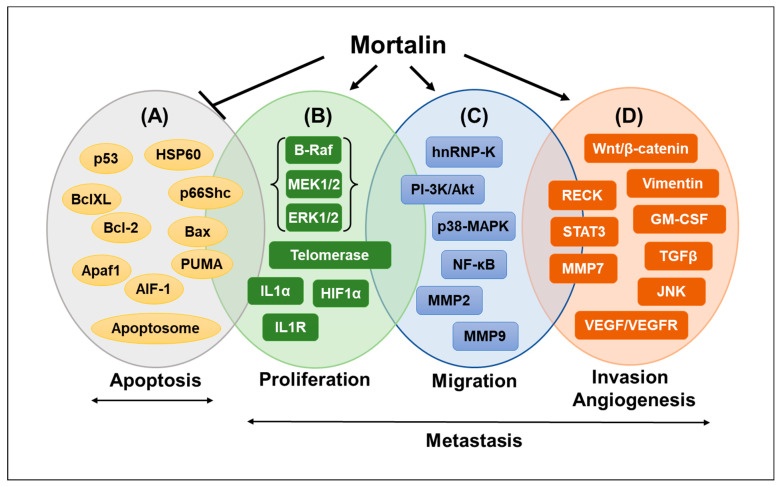
Role of Mortalin in tumorigenesis. (**A**) Mortalin blocks the extrinsic and intrinsic apoptotic pathways in multiple ways, including inhibition of anti-apoptotic activities of p53, BclXL, Bcl2, Hsp60, Apaf1, AIF-1, and promoting apoptotic functions of Bax and PUMA [58]. (**B**) Increase cell proliferation by upregulating B-Raf-MEK1/2-ERK1/2, telomerase, IL1α, and HIF-1α signaling [59]. (**C**) Promotion of cell migration by activating hnRNP-K, PI-3K/Akt, p38-MAPK, NF-kB, MMP2, and MM9 [60]. (**D**) Stimulation of invasion and angiogenesis by activating Wnt/β-catenin, RECK-STAT3-MMP, and TGFβ-JNK-VEGF signaling involved in tumor metastasis [61,62].

Mortalin interacts with p53 (commonly inactivated in most cancers), confining it to the cytoplasm and inactivating its nuclear transcriptional activation function, which is essential for the growth arrest of cells [42,43]. Under cellular stress, the anti-apoptotic protein Bcl-2 interacts with p53 and triggers a cascade of apoptotic signaling events independent of the nuclear transcriptional activation function of p53. Mortalin has been shown to interfere with p53/Bcl-2 interaction and thereby activates the anti-apoptotic axis [44,45], leading to the long-term persistence of cancer cells. Based on these findings, the inhibition of Mortalin–p53 interactions to restore the apoptotic function of p53 has been proposed as a feasible therapy for cancer [43]. The downregulation of Mortalin expression by its antisense expression induced senescence-like growth arrest in immortalized human cells [7]. Studies on Ras-Raf-activated tumors have demonstrated the critical involvement of Mortalin in the resistance of these tumors to apoptosis [46,47]. Mortalin can also enhance the activities of telomerase and heterogeneous ribonucleoprotein K (hnRNP-K), which are established hallmarks of malignantly transformed cancer cells [16]. Along with deactivating apoptotic signaling, cancer cells activate epithelial-to-mesenchymal transition (EMT), contributing to their continued survival and metastasis. Markers of activated EMT signaling have been well established and include cytoskeletal proteins involved in focal adhesion, PI3K–Akt, and JAK-STAT signaling, upregulation of mesenchymal markers like fibronectin (FN1), vimentin (VIM), CK14 (KRT14), β-catenin (CTNNB1), and hnRNP-K, and downregulation of epithelial markers like E-cadherin (CDH1), CK18 (KRT18), and CK8 (KRT8) [48,49,50,51]. Mortalin-enriched cancer cells have been shown to possess activated EMT, metastatic, angiogenic, cancer cell stemness, and drug resistance signaling, including activation of the Wnt/β-catenin pathway [15,52,53,54,55]. Some important molecular players and biological processes associated with Mortalin expression in cancer are shown in Figure 2. There have been multiple reports and studies wherein Mortalin has been suggested as an effective drug target for cancer [15,56,57]. In this premise, we review the existing Mortalin inhibitors and their chemical properties, biological mechanisms of action, and shortcomings.

## 3. Mortalin Inhibitors for Cancer Therapy

The known inhibitors of Mortalin can broadly be classified into inhibitory peptides, RNAs, and synthetic and natural small molecules.

### 3.1. Inhibitory Peptides

#### 3.1.1. UBX Domain-Containing Protein (UBXN2)

Ubiquitin-like (UBX) domain-containing protein, UBXN2A, has been shown to bind to Mortalin competitively, thereby inhibiting the p53–Mortalin interaction, rescuing p53 from the cytosolic sequestration and reactivating its tumor suppressor activity [63]. His-tag affinity pull-down assays and yeast two-hybrid studies showed that amino acid residues—Pro442, Lys555, and Ile558—in the SBD of Mortalin are critical for the interaction with UBXN2A [63]. Functional and biochemical assays confirmed that the overexpression of UBXN2A causes the abrogation of the Mortalin–p53 interaction, yielding induction of apoptosis as determined cell shrinkage, change in nuclear morphology, and detachment from the surface in colon cancer cells in culture and xenograft murine model [64].

#### 3.1.2. SMR-Derived Peptides

Secretion modification region (SMR) peptides, obtained from HIV-1 Nef protein, are effective against breast cancer [65]. Huang et al. (2019) further modified these peptides by (i) concatenating with a cell-penetrating peptide (CPP), SMR-CPP, (ii) adding an arginine-rich positively charged peptide from Tat protein of HIV-1, PEG-SMR, and (iii) adding a clusterin-binding peptide (CLU) that assists in the secretion of protein at the C-terminal of SMR [65,66]. Biochemical analyses of control and SMR-treated MDA-MB-231 and MCF-7 breast cancer and K562 leukemia cells revealed the inhibition of complement factor C9 and Mortalin [66]. Complement factor C9 is an established cancer marker [67]. Blocking the release of extracellular vesicle-containing complement proteins has been shown to cause cell death [68]. These SMR peptides also hindered the release of extracellular vesicles in cancer cells, thereby promoting complement-mediated cellular death [66]. The modified SMRs also caused a decrease in the expression of vimentin, a mesenchymal marker and a key regulator of EMT progression in metastatic cancers [65,66].

### 3.2. Inhibitory RNAs

#### miRNAs (miRNA-200b, mi-RNA-200c and miRNA217)

Complement-dependent cytotoxicity (CDC) is one of the numerous biological processes regulated by miRNAs and is well-studied in leukemia cells [69]. The C5b-9 complex is a key factor in CDC [70]. Mortalin helps protect the cells from the CDC by eliminating C5b-9 [27]. miRNA217, miR-200b, and miR200c binding and regulation sites were found in Mortalin mRNA [69]. Overexpressing either of the miRNAs (miRNA200b, miRNA200c, or miRNA217) caused a decrease in Mortalin mRNA [69]. On the other hand, cells exposed to inhibitors of these miRNAs showed upregulation of Mortalin mRNA [69]. These studies have suggested using miRNA-200b, mi-RNA-200c, and miRNA217 as Mortalin inhibitors in cancer treatment.

### 3.3. Synthetic Small Molecule Inhibitors of Mortalin

#### 3.3.1. MKT-077

**Figure 3:**
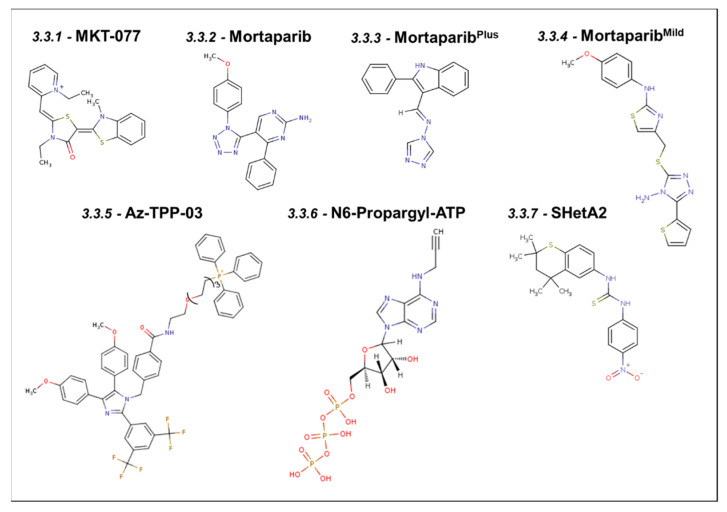
Synthetic small molecule inhibitors of Mortalin.

MKT-077, [IUPAC Name: 1-Ethyl-2-[[3-ethyl-5-(3-methyl-2(3H)-benzothiazolylidene)-4-oxo-2-thiazolidinylidene]methyl]-pyridinium chloride; chemical structure shown in Figure 3] was initially known to have an affinity for Hsp70 and actin in Ras-transformed cells [71,72]. It was then demonstrated to bind to Mortalin in a pull-down assay where MKT-077-conjugated Sepharose beads were used to pull out MKT-077 binding proteins. Probing these complexes with Mortalin-specific antibodies identified Mortalin as the binding partner, validated by immunoprecipitation experiments [7]. Subsequently, amino acid residues 252–310 of Mortalin are shown to bind to MKT-077 [7,73]. A stretch of those amino acid residues ranging from 252 to 283 was also shown to be involved in binding to p53, causing its cytoplasmic retention [73]. In agreement with these findings, MKT-077-treated cells showed abrogation of the Mortalin–p53 interaction, leading to the growth arrest of cancer cells, also marked by the upregulation of p21^WAF1^ expression, signifying the reactivation of transcriptional activation function of p53 [7]. High-potency MKT-077 derivatives, such as FJ-5826 and FJ-5744, have been shown to cause growth arrest at concentrations 10-fold lower than that of MKT-077. In MCF-7 cells, where p53 is inactivated due to its cytoplasm retention [74,75], treatment with 6.9 μM MKT-077, or FJ-5744 and FJ-5826, caused 10, 20, and 60-fold increases, respectively, in wild-type p53-dependent reporter activity assays [7]. Considering the mechanism of action of the MKT-077 and its derivatives, it was suggested that they would prove to be effective in the treatment of neuroblastoma, teratocarcinoma, and breast carcinoma, where p53 is inactivated largely by cytoplasmic sequestration. However, despite its efficacy, it failed in the clinical trial due to its adverse side effects and renal toxicity [76,77].

#### 3.3.2. Mortaparib

The mortaparib [IUPAC Name: 5-[1-(4-methoxyphenyl)(1,2,3,4-tetra azol-5-yl)]-4-phenylpyrimidine-2-ylamine] chemical structure, shown in Figure 3 [78], was isolated in a cancer cell-based screening assay with double readouts: (i) shift of Mortalin staining pattern from perinuclear to pancytoplasmic and (ii) nuclear translocation of p53 [78]. Indeed, mortaparib-treated cells showed a decrease in Mortalin–p53 binding and activation of tumor suppressor and apoptotic activities of p53 [79]. Furthermore, mortaparib caused the downregulation of Mortalin transcription and inactivated a major DNA repair enzyme (PARP-1). Based on these two criteria, the compound was named mortaparib. It promoted apoptosis in cancer cells, supported by upregulation of the pro-apoptotic BH3 proteins PUMA and NOXA [79]. The activation of tumor suppressor activity in p53 was supported by the upregulation of p21^WAF−1^, CDK4, pRb, E2F1, and CyclinD1 proteins [78]. These activities of mortaparib were demonstrated in a large variety of cancer cell lines, including cervical (HeLa), ovarian (SKOV3), prostate (PC3 and DU145), lung cancer (A549), colorectal adenocarcinoma (DLD1), non-small cell lung cancer (NCL-H1299), pancreatic carcinoma (SUIT-2), hepatoma (PLC), and human gastric cancer (MKN-45) [78]. Dose-dependent cytotoxic analyses showed that HeLa and SKOV3 showed stronger cytotoxicity [78]. Computational insights on mortaparib–PARP1 interaction revealed that it interacts with the carboxyl terminus of PARP1, which is also the region where Mortalin interacts [78]. Co-immunoprecipitation assays have supported the occurrence of Mortalin–p53–PARP-1 complexes and their abrogation by mortaparib, affecting DNA damage signaling in cancer cells. Furthermore, the transcriptional downregulation of Mortalin was connected to a reduction in N-cadherin, fibronectin, MMP3, MMP7, MMP9, MMP2, vimentin, and hnRNP-K (key proteins involved in the cell migration and metastatic characteristics of cancer cells [78].

#### 3.3.3. Mortaparib^Plus^

Mortaparib^Plus^ [IUPAC Name: 4-[(1E)-2-(2-phenylindol-3-yl)-1-azavinyl]-1,2,4-triazole; chemical structure shown in Figure 3] was also isolated in a cell-based screening as described for mortaparib. Two luminal-A breast cancer cell lines—MCF-7 (p53^wild type^) and T47D (p53^L194F^)—when treated with Mortaparib^Plus^, showed the transition of the perinuclear Mortalin staining pattern to the pancytoplasm, the relocated p53 to the nucleus, a decrease in Mortalin expression at the transcriptional level, and the inactivation of PARP-1 [80]. The reduced expression of Mortalin, yielding the reactivation of the transcriptional activity of wild-type p53, was recorded in MCF-7 cells. On the other hand, mutant p53 harboring T47D cells exhibited wild-type p53-independent mechanisms, including hyperactivation of PARP1 that was marked by the accumulation of PAR polymer and decreased ATP levels. Of note, the complete abrogation of the AIF–Mortalin complexes (enriched in T47D cells), apoptosis, or PAR–Thanatos was not observed, suggesting the limited activity of Mortaparib^Plus^ in cell lines of similar nature [79,80]. Further, a study in colorectal cancer cells, HCT116 (wild-type) and DLD-1 (mutant p53), demonstrated the abrogation of Mortalin–p53 complex formation in Mortaparib^Plus^-treated cells, increasing the levels of p53 mRNA and decreasing the expression of Mortalin, leading to growth arrest/apoptosis. An increment in the pro-apoptotic markers, such as BAX, PUMA, and cleaved Caspase3, and a decrease in the anti-apoptotic proteins, such as Bcl-xl, Caspase9, Caspase7, and Caspase3, marked the increased proportion of apoptotic cells in Mortaparib^Plus^-treated cultures. Cells undergoing growth arrest in the G2/M phase showed increased p21WAF1 and decreased CDK4, Cyclin D1, and E2F1 proteins. Similar to the breast cancer cells, the colorectal cancer cells showed inhibition of PARP1 function and accumulation of DNA damage [81]. In addition, these cells showed (i) upregulation of tumor suppressor proteins p73 and (ii) downregulation of CARF, which is super-enriched in many malignant cancers. Collectively, these studies have demonstrated multiple mechanisms of cytotoxicity of Mortaparib^Plus^ to cancer cells, and based on the stronger anti-cancer activity, the compound was hence called Mortaparib^Plus^. Mortaparib^Plus^, but not mortaparib, was shown to cause the downregulation of ACE2 and TMPRSS2 receptors involved in SARS-CoV-2 infection. In silico analyses supported these data, showing that only Mortaparib^Plus^ stably binds into the catalytic pocket of TMPRSS2 [82].

#### 3.3.4. Mortaparib^Mild^

Mortaparib^Mild^ [IUPAC Name: 4-[(4-amino-5-thiophen-2-yl-1,2,4-triazol-3-yl)sulfanylmethyl]-N-(4-methoxyphenyl)-1,3-thiazol-2-amine, chemical structure shown in Figure 3] is the third molecule identified as a co-inhibitor of Mortalin and PARP1. Although required at high concentrations as compared to the described mortaparib and Mortaparib^Plus^, it has been shown to cause cytotoxicity in the wild-type p53 (HCT116) cells as well as p53-null (Saos-2 and SKOV3) cells through p53-dependent and -independent pathways, respectively. The cells decreased Mortalin and PARP1 levels, affecting mitochondrial functions and DNA damage repair signaling [83]. As supported by phenotypic and molecular marker analyses, Mortaparib^Mild^-treated cancer cells showed compromised migration, invasion, and cluster formation, essential characteristics of cancer cells involved in metastasis, to which Mortalin has been shown to contribute [47,62,83,84]. In light of these functions of Mortaparib^Mild^ and taking advantage of the stronger cytotoxicity of Mortaparib^Plus^, combinations consisting of different ratios of three mortaparibs (Mortaparib^Mix-AP^, Mortaparib^Mix-AM^, and Mortaparib^Mix-AS^) have been suggested for enhanced anti-proliferation, anti-migration, and anti-stress activities [85].

**Figure 4:**
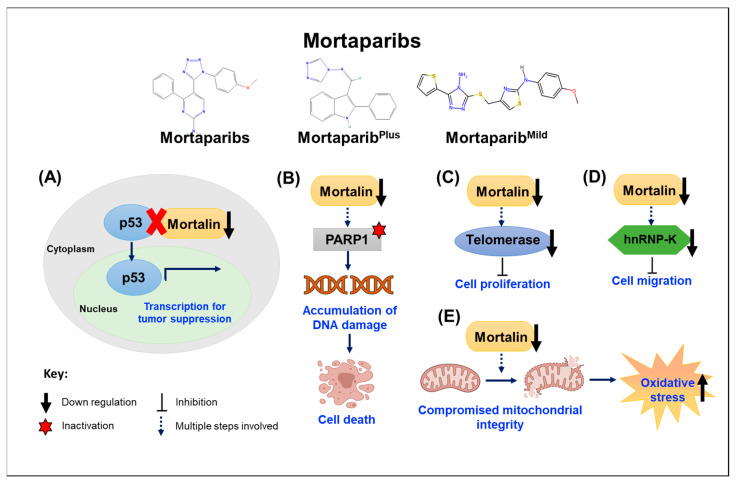
Mortaparib(s) mediate the anti-cancer response through various mechanisms: (**A**) abrogation of Mortalin–p53 interaction, resulting in nuclear translocation and reactivation of p53 function; (**B**) inhibition of Mortalin–PARP1 interaction and DNA repair; (**C**) downregulation of telomerase function and inhibition of cell proliferation; (**D**) downregulation of Mortalin and compromised hnRNP-K function and cell migration; (**E**) downregulation of Mortalin and compromised mitochondrial integrity and increase in oxidative stress.

A schematic representing the mechanisms of action of mortaparib(s) is shown in Figure 4.

#### 3.3.5. Apoptozole–Triphenylphosphonium Conjugate (Az-TPP-03)

Az-TPP-O3 is a conjugate of Az–Triphenylphosphonium that inhibits the mitochondrial Mortalin’s ATPase activity, thereby initiating apoptosis in tumor cells [86]. The chemical structure of Az-TPP-O3 is shown in Figure 3. The TPP moiety is an established motif that targets mitochondria [87]. When bound to Az-TPP-03, mitochondrial Mortalin could not generate monophosphate from ATP, as exhibited in a PiColorLock colorimetric assay [86]. A cell viability assay performed on 20 different human cancer cell lines (HeLa, MIA-Paca2, Capan-1, As-PC1, CFPAC-1, HCT116, HT29, SK-OV-3, OVCAR3, K562, HL60, PLC, HepG2, U87, U373, A549, H226, PC3, LNCAP, and DU145) showed that it is highly effective towards a broad spectrum of cancer cells (IC_50_ value was within 0.5–1.5 µM) [86]. Mitochondrial Mortalin suppresses apoptosis by directly binding to p66Shc and p53 in cancer cells [57,88]. Az-TPP-03-bound mitochondrial Mortalin caused an increase in apoptosis in cancer cells, confirmed by an increase in the uptake of annexin V and PI [89] and cell shrinkage [86], increased fragmentation of DNA [86], and increased pro-apoptotic members (Bax or Bak) of the Bcl-2 family of proteins [90].

#### 3.3.6. ADP Analog Inhibitors

N6-propargyl ADP, an analog of ADP, was tested as a potential cell suicide inhibitor. It was found that the modified nucleotides at the N6-position and the 2-position of the adenosine group can effectively interact with Mortalin-NBD, thus inhibiting its ATPase activity [91]. The chemical structure of N6-propargyl ADP is shown in Figure 3. Accordingly, ATP hydrolysis inhibition studies were done on modified ADP nucleotides, which included 6-Bn ADP, 2-Cl ADP, 6-PheEt ADP, 2-MeS ADP, 6-Fu ADP, 6-(3-MeBn) ADP, and 6-cHe ADP. All these compounds were found to be potent inhibitors. 2-Cl ADP, 6-PheEt ADP, and 6-Bn ADP demonstrated higher potential to inhibit ATP hydrolysis than unmodified ADP. Maximum inhibition was shown by 2-Cl ADP, with a Ki^app^ of 45.05 μM. It also exhibited the strongest affinity with the Mortalin–NBD site compared to the other ADP analogs [91]. The Ki^app^ and log IC_50_ values of these compounds, as obtained in the study, are shown in Table 1 [91].

#### 3.3.7. SHetA2

SHetA2 [IUPAC Name: 1-(4-nitrophenyl)-3-(2,2,4,4-tetramethyl-3H-thiochromen-6-yl)thiourea; chemical structure shown in Figure 3] is a flexible heteroarotinoid (Flex-Het) with cancer therapeutic properties [92,93]. In a study to determine the interacting partners of SHetA2, Mortalin was found bound with SHetA2-conjugated magnetic microspheres when the protein extract of the ovarian cancer cell line was tested [93]. Later on, co-immunoprecipitation studies reported that SHetA2 perturbs the binding of Mortalin to p53 and p66shc (Src homologous–collagen homolog) in cancer cells [93]. The study also revealed that SHetA2 promotes a drop in mitochondrial membrane potential and mitochondrial swelling, which further causes the release of cytochrome c, generation of reactive oxygen species (ROS), and activation of the intrinsic apoptosis pathway activation [92,94,95,96,97,98,99], thus implying that SHetA2 acts by inhibiting Mortalin. SHetA2-mediated release of p66shc from Mortalin causes mitochondrial pore opening, cytochrome c release, and ROS generation [100]. Another study on focal ischemia reported that the overexpression of Mortalin resulted in a reduction in ROS and protection from ischemic injury in both in vivo and in vitro models of the disease [37,101].

## 4. Natural Small Molecule Inhibitors of Mortalin 

Natural products are believed to be safer, with fewer side effects, when compared to synthetic drugs and their derivatives. Various compounds extracted from natural resources like plants, animals, or even microorganisms have a long-recorded history of being used to treat various ailments, including cancer. Several studies have reported natural compounds extracted from various sources as inhibitors of the Mortalin–p53 interaction, thereby reactivating the transcriptional activation function of the latter [57,62]. For example, alcoholic extracts from Ashwagandha (*Withania somnifera*) contain alkaloids like Withaferin-A and Withanone, which have been shown to block the Mortalin–p53 interaction [7,11]. Some examples of such compounds with the potential to inhibit Mortalin function in cancer are as follows.

### 4.1. Experimentally Validated Natural Inhibitors of Mortalin

#### 4.1.1. Fucoxanthin

**Figure 5:**
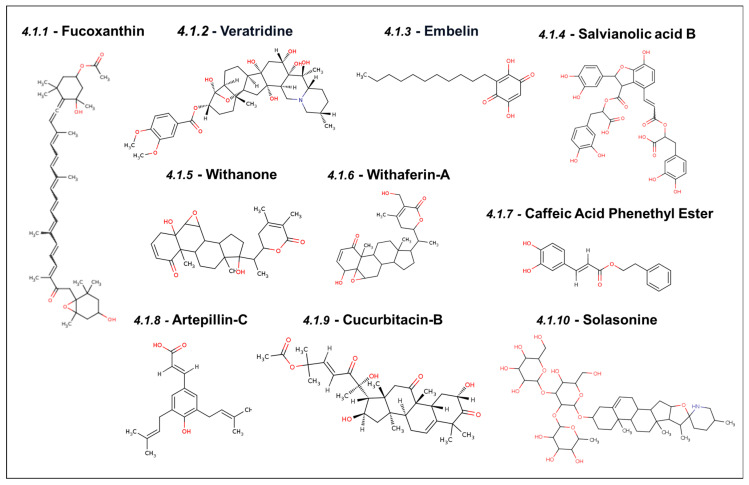
Compounds from natural sources as inhibitors of Mortalin.

Fucoxanthin is a natural carotenoid extracted from edible brown algae (*Undaria pinnatifida*), rich in β-carotene known for its anti-cancer and anti-stress properties (structure shown in Figure 5) [102]. It has been shown to induce growth arrest at the G0-G1 phase at low doses and apoptosis at high doses in human bladder cancer T24 cells. The cell cycle arrest was shown to be mediated by the upregulation of p21^WAF1^ and the downregulation of CDK-2, CDK-4, cyclin D1, and cyclin E. Apoptosis at high dose was the result of the abrogation of the Mortalin–p53 interaction that reactivated p53 function in the nucleus, followed by the upregulation of cleaved caspase-3 [103]. Yet another study demonstrated that fucoxanthin was selectively cytotoxic to cancer cells by inhibiting the interaction between Mortalin–p53 in the cytoplasm. It was also shown to cause transcriptional downregulation of Mortalin. Furthermore, as a consequence, many other proteins were found to be downregulated. These included proliferation-associated proteins (STAT3, pSTAT3, RB, and pRB), survival protein (Survivin), stemness-related proteins (Wnt-1 and β-catenin), EMT markers (fibronectin, MMP2, and vimentin), and angiogenesis factor (VEGF). Fucoxanthin inhibited cancer cells’ migration, invasion, and angiogenic characteristics [9].

#### 4.1.2. Veratridine

Veratridine is an alkaloid derived from the Liliaceae plant family, as shown in Figure 5. It has been shown to possess anti-tumor activity mediated by the selective enhancement of the transcription of UBXN2A, a binding partner and inhibitor of Mortalin [104]. Veratridine was shown to inhibit Mortalin indirectly by upregulating UBXN2A. Doxycycline-induced UBXN2A expression increased apoptotic markers, such as p21^WAF−1^, PARP-1, and caspase3. An in vivo study showed a 50% reduction in tumor size in a xenograft mouse model treated with UBXN2A. Veratridine was also shown to yield synergetic effects in combination with two other chemotherapeutic drugs, 5-FU and etoposide [104].

#### 4.1.3. Embelin

Embelin is a naturally occurring benzoquinone (shown in Figure 5), a phenolic compound present in fruits of *Embelia ribes* that is commonly used as an anti-inflammation, anti-fever, antibacterial, and anti-cancer agent in Indian traditional medicine (Ayurveda) [105]. Cancer cells treated with low dosages of embelin show reduced levels of Mortalin with an increase in tumor suppressor p53 and ROS, leading to growth arrest. This abrogation of Mortalin–p53 was shown using molecular docking, followed by its validation by immunostaining of the two proteins in control and embelin-treated cells. The latter showed a decrease in Mortalin at the mRNA and protein levels. Along with higher levels of ROS, embelin-treated cells exhibited a low expression of proteins associated with cell proliferation and differentiation characteristics like TGF-β1, PDGF-A, IGF-1, IGF-II, KIT, KDR, CSF2, and PIGF. Also, they had a reduced expression of metastasis-regulatory proteins like MMPs, vimentin, β-catenin, TGF-β, and Wnt-3a. A high dose of embelin, on the other hand, caused cell apoptosis with a decrease in the expression levels of Bcl2 and pro-PARP [57].

#### 4.1.4. Salvianolic Acid B

Salvianolic acid B (Sal-B) is a phenolic compound formed by mixing three compounds—two extracted from *Salvia miltiorrhiza* (Danshen) and caffeic acid. It is known for its anti-oxidative activity and is commonly used in ancient Chinese traditional medicine [106]. The chemical structure of Sal-B is shown in Figure 5. Teng et al. showed that Mortalin promotes the migration and invasion of hepatocellular carcinoma by regulating the RECK/STAT pathway [41]. RECK (reversion-inducing cysteine-rich protein with Kazal motifs) is a glycoprotein present in the cell membrane, and its expression is highly reduced in tumor cells. An inverse correlation between the levels of RECK protein and Mortalin expression was established. The upregulation of RECK protein prevents the migration and invasion of cancer cells in hepatocellular carcinoma by downregulating the downstream protein STAT3 and matrix metalloproteinases like MMP2, MMP9, and MT1-MMP. An in silico approach was used to demonstrate that Sal-B and two other caffeic acid derivatives stably bind to the p53 binding site of Mortalin. Sal-B can specifically bind with Mortalin and induce proteasomal degradation. Western blot analysis further confirmed that Sal-B-treated cells have high E-Cadherin and RECK, while STAT3, N-Cadherin, Mortalin, and vimentin levels were reduced. Thus, it was concluded that Sal-B prevents migration and cell invasion by the degradation of Mortalin, leading to dysregulation of the RECK/STAT3 pathway [62].

#### 4.1.5. Withanone

Withanone is one of the primary constituents of the extract of Ashwagandha (*Withania somnifera*), a tropical herb regularly used in Indian traditional medicine [107]. The chemical structure of Withanone is shown in Figure 5. As demonstrated by computational and experimental studies, it is known for its anti-cancer activity mediated through multiple molecular targets, including Mortalin. Withanone was shown to bind to Mortalin, disrupt Mortalin–p53 binding, and induce the translocation and tumor suppressor function of p53 in the nucleus. Of note, it was seen to bind to the amino acid residues of Mortalin that are also involved in binding to p53 and MKT-077; the docking affinity of Withanone to Mortalin was comparable to that of MKT-077. These interactions have been validated by immunoprecipitation analyses in cancer cells [7].

#### 4.1.6. Withaferin-A

Withaferin-A is another primary constituent of Ashwagandha extract. Treatment of cancer cells with withaferin-A caused a shift in the Mortalin staining pattern from perinuclear to pancytoplasmic, indicating the abrogation of the Mortalin–p53 interaction. Further, molecular docking has shown that withaferin-A interacts with the p53-binding region of Mortalin. Low doses of withaferin-A have been shown to arrest cell growth, with increased p21^WAF1^ that localizes in the nucleus. On the other hand, high doses of withaferin-A-induced apoptosis, mediated by a p21^WAF1^-independent additional mechanism as p21^WAF1^, were found to be downregulated. Withaferin-A-treated cells show a reduction in Mortalin, affecting cancer cell characteristics in multiple ways. In contrast to Withanone, withaferin-A is effective in low concentrations and is toxic to normal cells [11]. The anti-cancer activity of withaferin has been supported by many studies, demonstrating its multiple mechanisms of action [107]. The chemical structure of withaferin-A is shown in Figure 5.

#### 4.1.7. Caffeic Acid Phenethyl Ester

Caffeic Acid Phenethyl Ester (CAPE) is a crucial chemical constituent of New Zealand propolis and is known for its various health benefits. CAPE was tested for its anti-cancer activity in various human cancer cell lines and was cytotoxic to all, with an IC_50_ in the range of 5–100 μM. The chemical structure of CAPE is shown in Figure 5. Depending upon the dose, CAPE-treated cells show growth arrest or apoptosis by the upregulation of the p53 function due to the abrogation of Mortalin–p53 interactions. Using a molecular docking approach, it was shown to possess an excellent binding affinity for the p53-binding domain of Mortalin. It caused the downregulation of Mortalin at both protein and transcript levels (thereby further downregulating multiple targets involved in cell migration (vimentin, MMP-2, MMP-3, β-catenin, TGF-β, and WNT-3a). The anti-cancer activity of CAPE has also been validated in in vivo tumor assays in nude mice [57]. Additionally, a combination of withaferin-A and CAPE has been reported to offer selective toxicity and better potency to cancer cells. A combination of low nontoxic concentrations of wi-A and CAPE was shown to effectively inhibit migration and the metastatic characteristics of cancer [57,108].

#### 4.1.8. Artepillin-C

Artepillin-C is a major bioactive ingredient of Brazilian green propolis. Like CAPE, Artepillin-C abrogates Mortalin–p53 complex formation, causing the translocation and activation of p53 in the nucleus and yielding growth arrest in cancer cells. Artepillin-C interacted at the p53-binding interface of Mortalin with a docking score of −6.99 kcal/mol. Although required at a high concentration compared to CAPE and withaferin-A, it was shown to interfere with the Mortalin–p53 interaction and reactivated the tumor suppressor activity of the latter [57]. Low concentrations of Artepillin-C were seen to possess anti-stress potential [57]. The chemical structure of Artepillin-C is shown in Figure 5.

#### 4.1.9. Cucurbitacin-B

Cucurbitacin-B ((19-(10→9β)-abeo-10-lanost-5-ene triterpene or Cuc-B; chemical structure shown in Figure 5) is a variant of the biochemical class of Cucurbitacin that is present in plants of the Cucurbitaceae family, including gourds and pumpkins. It is a secondary metabolite, has a triterpene structure, and is synthesized to defend the plant from herbivores [109]. A recent study observed that Cuc-B can bind to the p53-binding site of Mortalin with a binding affinity of −5.54 kcal/mol, as confirmed by docking studies. Interestingly, the affinity of Cuc-B was far greater than that of MKT-077, which was −1.99 kcal/mol. Further, its effect on HDM2 (a p53 antagonist enriched in cancer cells and a regulator of cancer cell proliferation and migration characteristics) was also investigated. The interaction of Cuc-B with HDM2 was similar to the mode adopted by Y30, a well-known inhibitor of HDM2. Molecular analyses showed that Cuc-B downregulates several proteins involved in modulating the process of cell migration [10]. A novel combination of Cuc-B with Withanone demonstrating higher anti-cancer activity, selective cytotoxicity, and anti-metastatic potential has also been reported. This combination targeted the Mortalin–p53 interaction and the hnRNP-K protein and triggered replicative senescence, inhibiting tumor progression and metastasis in vivo [57].

#### 4.1.10. Solasonine

In silico screening of natural compounds recognized solasonine from the Solanaceae family, a steroidal glycoalkaloid (Figure 5), as a potential inhibitor of the Mortalin–p53 interaction. The docking of solasonine with the PDB structure of Mortalin and p53 showed non-covalent interactions between solasonine and the residues lying within the interacting region of the proteins. In HepG2 cells, solasonine treatment led to dose-dependent apoptosis, attributed to the abrogation of the Mortalin–p53 interaction in the cytoplasm and its translocation to the nucleus for its transcriptional activity. The increased expression of free p53 was also associated with an increased expression of p21^WAF−1^ and cleavage products of nuclear lamin B1 and PARP protein. Solasonine was also found effective in the p53-deficient cell line Hep3b, suggesting an alternative mechanism that is p53-independent but yet to be elucidated here [110].

### 4.2. Natural Inhibitors of Mortalin Predicted Using Computer-Aided Drug Discovery

#### 4.2.1. Curzerenone

**Figure 6:**
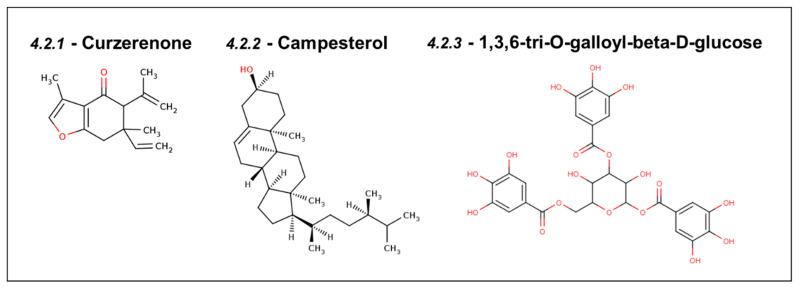
Compounds from natural sources as inhibitors of Mortalin.

*Curcuma zedoaria*, a plant species belonging to the Zingiberaceae family, is known for its traditional use against various ailments, including cervical, breast, and colorectal cancers [111]. Curzerenone derived from this plant has been demonstrated to dock in the p53-binding site of Mortalin with a docking score (−7.4 kcal/mol) better than the known Mortalin drugs, such as MKT-077 and CAPE. The chemical structure of Curzerenone is shown in Figure 6. The binding pose of Curzerenone was also similar to the control compounds, MKT-077 and CAPE [112].

#### 4.2.2. Campesterol

Campesterol is a phenolic compound derived from black rice (*Oryza sativa* L.) that regulates the cell cycle and induces apoptosis. The chemical structure of campesterol is shown in Figure 6. In a study, sixteen compounds extracted from black rice were screened using molecular docking, and it was found that campesterol binds well with a homology-modeled structure of Mortalin at the p53 binding site. The docking of p53 with Mortalin proteins showed decreased attractiveness and increased repulsive energies upon the introduction of campesterol in the binding site [113].

#### 4.2.3. DTOM (1,3,6-tri-O-galloyl-beta-D-glucose)

The high-throughput screening of natural compounds from the ZINC database against Mortalin protein identified DTOM ((2R, 3R, 4S, 5R, 6S)-3,5- dihydroxy-4,6-bis[(3,4,5 trihydroxy benzoyl) oxy]tetrahydro- pyran-2-yl]methyl; chemical structure shown in Figure 6) and its analog TTOM ([2-[(2S,3R,4S,5S,6R) -3,4,5-trihydroxy -6(hydroxymethyl) te-trahydropyran-2yl] oxyphenyl] methyl) as potential hits. Both of these natural compounds could interact stably with the p53-binding site of Mortalin, consisting of amino acid residues 253–282. The MD simulations were run for 20 ns to further assess the binding stability with Mortalin for both of these natural compounds from *Euphorbia lunulata* [114].

## 5. Inhibitors of the Hsp70 Family of Proteins—Non-Specific Inhibitors of Mortalin

As mentioned above, Mortalin belongs to the Hsp70 chaperone family of proteins. Therefore, non-specific inhibitors designed against Hsp70 proteins (Table 2) can potentially inhibit the functional activity of Mortalin, as well. Broad-spectrum Hsp70 inhibitors like JG-98 and Quercetin (3,3′,4′,5,7-pentahydroxyflavone) have already been shown to inhibit Mortalin and are, hence, worth mentioning here. However, the non-specific inhibition of Hsp70 proteins in cancer may lead to various unwanted effects, warranting a debate over their use in such cases.

## 6. Conclusions

Mortalin plays a significant role in various cellular processes, including proliferation, protein homeostasis, neurodegeneration, and viral infections [7,15,31,56,57,84,135,136,137,138,139,140]. Its critical involvement in oncogenesis makes it a promising candidate target for cancer drug development. The current review highlights the potential of synthetic and natural compounds to abrogate the cancer-promoting functions of Mortalin, offering hope for more effective cancer therapies. However, more elaborate studies are required to assess these compounds’ toxicity, specificity, and safe delivery and to explore their full therapeutic potential in the clinic.

## Figures and Tables

**Table 1:** The Ki^app^ and log IC_50_ values of ADP analog inhibitors.

ADP Analog	Structure	K_i_^app^(µM)	Log IC_50_
ADP	** 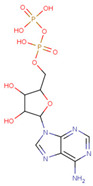 **	352.90 ± 36.21	2.613 ± 0.065
2-Cl ADP	** 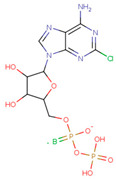 **	45.05 ± 2.42	1.752 ± 0.037
6-Bn ADP	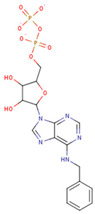	86.51 ± 10.26	2.074 ± 0.084
6-PheEt ADP	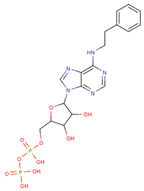	97.29 ± 9.96	2.238 ± 0.041
6-Fu ADP	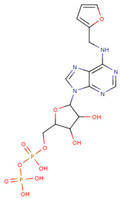	564.60 ± 49.63	2.747 ± 0.071
2-MeS ADP	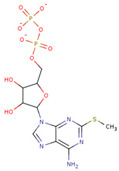	523.20 ± 98.35	2.955 ± 0.169
6-(3-MeBn)ADP	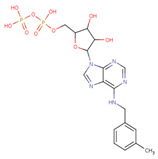	675.30 ± 99.35	3.154 ± 0.124
6-cHe ADP	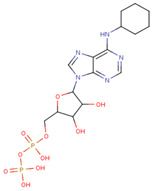	733.10 ± 82.07	3.203 ± 0.082

**Table 2:** Non-specific inhibitors of the Hsp70 family of proteins.

Inhibitor	Mode of Action	Reference
Small Compound Inhibitors		
VER-155008	Derived from adenosine, it competitively inhibits the ATPase activity of Hsp70. It also regulates allosterically the interaction between SBD and NBD.	[115]
Gold Nanorods -formulated MPEG-AuNR -VER-155008	VER-155008, along with gold nanorods, increases the photosensitivity of tumor cells, and VER-155008 micelles with gold nanorods enhance the inhibition of Hsp70.	[116]
NSC 630668-R/1	R/1 is known to inhibit the DnaJ-mediated ATP hydrolysis activity of Hsp70. It is also known to block the Hsp70-stimulated translocation of its pre-protein into the microsomal vesicles derived from yeast.	[117]
MAL3-101	MAL3-101 inhibits Hsp70 allosterically by inhibiting ATPase activity of Hsp70.	[118]
DMT3132	DMT3132 is a MAL3-101 analog that inhibits Hsp70 by inhibiting its ATPase activity.	[119]
DMT3024	DMT3024 is known to affect the ATPase activity of Hsp70.	[119]
MAL2-11B	Inhibits Hsp70 ATPase activity by suppressing the J domain.	[120]
Pifithrin-μ (2-phenylethynesulfonamide)	Interacts with the SBD and disrupts interactions with partners such as Hsp40, p53, and p62.	[121,122]
YM-1	An MKT-077 derivative with better cytosolic localization; inhibits BAG3 interaction with Hsp70.	[123,124]
JG-83	An MKT-077 analog; binds at a similar site and hinders interactions with cochaperones and other protein partners.	[125]
JG-84	An MKT-077 derivative that interacts with the allosteric DnaK, NBD of Hsp70, thereby disrupting ATP hydrolysis and affecting the chaperone function.	[125]
JG-98	Abrogation of Hsp70 and the Bcl-2 associated anthanogene3 (Bag3) protein–protein interaction. Also inhibits Mortalin function.	[123,126]
Methylene blue	Inhibits the chaperone function of the Hsp70 protein by binding with the glucocorticoid receptor.	[127,128]
Natural Inhibitors		
15-deoxyspergualin (DSG)	Inhibits the ATPase domain of Hsp70 by interacting with the EEVD domain targeting the ABD.	[120]
Piericidin A	Suppresses GRP78.	[129]
Verrucosidin	Suppresses GRP78. It also inhibits the electron transport chain complex I.	[129]
Epigallocatechin-3-gallate (EGCG)	Blocks the ATPase domain of GRP78, thereby repressing it.	[130]
Quercetin (3,3′,4′,5,7-pentahydroxyflavone)	Inhibits the activation of Hsp70 at transcription stage via preventing Hsf1 activation. Also inhibits Mortalin function.	[120]
Nano-formulated Quercitin (Q-PEGL)	Quercetin on liposomal pegylation improves the bioavailability and solubility of quercetin. The exact mechanism of quercetin activity is unknown.	[131]
Kahweol	Attenuates expression of Hsp70, although the mechanism is unknown.	[132]
Cantharidin	Inhibits interaction of Hsp70 promoter and Hsf1, consequently blocking Hsf1-mediated expression of Hsp70.	[133]
Docosahexaenoic acid (DHA)	Downregulation of GRP78 protein by modulating upstream pERK1/2 and activation of caspase-4-mediated apoptosis.	[134]

## References

[1] Daugaard M., Rohde M., Jäättelä M. (2007). The heat shock protein 70 family: Highly homologous proteins with overlapping and distinct functions. FEBS Lett..

[2] Mayer M.P., Bukau B. (2005). Hsp70 chaperones: Cellular functions and molecular mechanism. Cell. Mol. Life Sci..

[3] Radons J. (2016). The human HSP70 family of chaperones: Where do we stand?. Cell Stress Chaperones.

[4] Wadhwa R., Kaul S.C., Ikawa Y., Sugimoto Y. (1993). Identification of a novel member of mouse hsp70 family. Its association with cellular mortal phenotype. J. Biol. Chem..

[5] Ran Q., Wadhwa R., Kawai R., Kaul S.C., Sifers R.N., Bick R.J., Smith J.R., Pereira-Smith O.M. (2000). Extramitochondrial localization of mortalin/mthsp70/PBP74/GRP75. Biochem. Biophys. Res. Commun..

[6] Bhattacharyya T., Karnezis A.N., Murphy S.P., Hoang T., Freeman B.C., Phillips B., Morimoto R.I. (1995). Cloning and Subcellular Localization of Human Mitochondrial hsp70 (∗). J. Biol. Chem..

[7] Kaul S.C., Wadhwa R. (2012). Mortalin Biology: Life, Stress and Death.

[8] Yang H., Zhou X., Liu X., Yang L., Chen Q., Zhao D., Zuo J., Liu W. (2011). Mitochondrial dysfunction induced by knockdown of mortalin is rescued by Parkin. Biochem. Biophys. Res. Commun..

[9] Garg S., Afzal S., Elwakeel A., Sharma D., Radhakrishnan N., Dhanjal J.K., Sundar D., Kaul S.C., Wadhwa R. (2019). Marine carotenoid fucoxanthin possesses anti-metastasis activity: Molecular evidence. Mar. Drugs.

[10] Huifu H., Shefrin S., Yang S., Zhang Z., Kaul S.C., Sundar D., Wadhwa R. (2024). Cucurbitacin-B inhibits cancer cell migration by targeting mortalin and HDM2: Computational and in vitro experimental evidence. J. Biomol. Struct. Dyn..

[11] Widodo N., Priyandoko D., Shah N., Wadhwa R., Kaul S.C. (2010). Selective killing of cancer cells by Ashwagandha leaf extract and its component Withanone involves ROS signaling. PLoS ONE.

[12] Roy A., Kucukural A., Zhang Y. (2010). I-TASSER: A unified platform for automated protein structure and function prediction. Nat. Protoc..

[13] Kampinga H.H., Hageman J., Vos M.J., Kubota H., Tanguay R.M., Bruford E.A., Cheetham M.E., Chen B., Hightower L.E. (2009). Guidelines for the nomenclature of the human heat shock proteins. Cell Stress Chaperones.

[14] Vishwanathan V., D’Silva P. (2022). Loss of function of mtHsp70 Chaperone variants leads to mitochondrial dysfunction in congenital sideroblastic anemia. Front. Cell Dev. Biol..

[15] Esfahanian N., Knoblich C.D., Bowman G.A., Rezvani K. (2023). Mortalin: Protein partners, biological impacts, pathological roles, and therapeutic opportunities. Front. Cell Dev. Biol..

[16] Ryu J., Kaul Z., Yoon A.R., Liu Y., Yaguchi T., Na Y., Ahn H.M., Gao R., Choi I.K., Yun C.O. (2014). Identification and functional characterization of nuclear mortalin in human carcinogenesis. J. Biol. Chem..

[17] Wiedemann N., Pfanner N. (2017). Mitochondrial machineries for protein import and assembly. Annu. Rev. Biochem..

[18] Schilke B., Williams B., Knieszner H., Pukszta S., D’Silva P., Craig E.A., Marszalek J. (2006). Evolution of mitochondrial chaperones utilized in Fe-S cluster biogenesis. Curr. Biol..

[19] D’Eletto M., Rossin F., Occhigrossi L., Farrace M.G., Faccenda D., Desai R., Marchi S., Refolo G., Falasca L., Antonioli M. (2018). Transglutaminase Type 2 Regulates ER-Mitochondria Contact Sites by Interacting with GRP75. Cell Rep..

[20] Liu Y., Ma X., Fujioka H., Liu J., Chen S., Zhu X. (2019). DJ-1 regulates the integrity and function of ER-mitochondria association through interaction with IP3R3-Grp75-VDAC1. Proc. Natl. Acad. Sci. USA.

[21] Szabadkai G., Bianchi K., Várnai P., De Stefani D., Wieckowski M.R., Cavagna D., Nagy A.I., Balla T., Rizzuto R. (2006). Chaperone-mediated coupling of endoplasmic reticulum and mitochondrial Ca2+ channels. J. Cell. Biol..

[22] Fiorese C.J., Schulz A.M., Lin Y.F., Rosin N., Pellegrino M.W., Haynes C.M. (2016). The Transcription Factor ATF5 Mediates a Mammalian Mitochondrial UPR. Curr. Biol..

[23] Smyrnias I. (2021). The mitochondrial unfolded protein response and its diverse roles in cellular stress. Int. J. Biochem. Cell Biol..

[24] Gancz D., Fishelson Z. (2009). Cancer resistance to complement-dependent cytotoxicity (CDC): Problem-oriented research and development. Mol. Immunol..

[25] Mazkereth N., Rocca F., Schubert J.R., Geisler C., Hillman Y., Egner A., Fishelson Z. (2016). Complement triggers relocation of Mortalin/GRP75 from mitochondria to the plasma membrane. Immunobiology.

[26] Pilzer D., Saar M., Koya K., Fishelson Z. (2010). Mortalin inhibitors sensitize K562 leukemia cells to complement-dependent cytotoxicity. Int. J. Cancer.

[27] Ray M.S., Moskovich O., Iosefson O., Fishelson Z. (2014). Mortalin/GRP75 binds to complement C9 and plays a role in resistance to complement-dependent cytotoxicity. J. Biol. Chem..

[28] Resendez E., Attenello J.W., Grafsky A., Chang C.S., Lee A.S. (1985). Calcium ionophore A23187 induces expression of glucose-regulated genes and their heterologous fusion genes. Molecular Cell. Biol..

[29] Kögel D., Schomburg R., Copanaki E., Prehn J.H.M. (2005). Regulation of gene expression by the amyloid precursor protein: Inhibition of the JNK/c-Jun pathway. Cell Death Differ..

[30] Jin J., Hulette C., Wang Y., Zhang T., Pan C., Wadhwa R., Zhang J. (2006). Proteomic Identification of a Stress Protein, Mortalin/mthsp70/GRP75: Relevance to Parkinson Disease*S. Mol. Cell Proteom..

[31] Texier B., Prime M., Atamena D., Belenguer P., Szelechowski M. (2023). Mortalin/Hspa9 involvement and therapeutic perspective in Parkinson’s disease. Neural Regen. Res..

[32] Franco-Iborra S., Vila M., Perier C. (2018). Mitochondrial quality control in neurodegenerative diseases: Focus on Parkinson’s disease and Huntington’s disease. Front. Neurosci..

[33] Kalmar B., Greensmith L. (2017). Cellular chaperones as therapeutic targets in ALS to restore protein homeostasis and improve cellular function. Front. Neurosci..

[34] Royer-Bertrand B., Castillo-Taucher S., Moreno-Salinas R., Cho T.J., Chae J.H., Choi M., Kim O.H., Dikoglu E., Campos-Xavier B., Girardi E. (2015). Mutations in the heat-shock protein A9 (HSPA9) gene cause the EVEN-PLUS syndrome of congenital malformations and skeletal dysplasia. Sci. Rep..

[35] Schmitz-Abe K., Ciesielski S.J., Schmidt P.J., Campagna D.R., Rahimov F., Schilke B.A., Cuijpers M., Rieneck K., Lausen B., Linenberger M.L. (2015). Congenital sideroblastic anemia due to mutations in the mitochondrial HSP70 homologue HSPA9. Blood. J. Am. Soc. Hematol..

[36] Qu M., Zhou Z., Xu S., Chen C., Yu Z., Wang D. (2011). Mortalin overexpression attenuates beta-amyloid-induced neurotoxicity in SH-SY5Y cells. Brain Res..

[37] Xu L., Voloboueva L.A., Ouyang Y., Giffard R.G. (2009). Overexpression of mitochondrial Hsp70/Hsp75 in rat brain protects mitochondria, reduces oxidative stress, and protects from focal ischemia. J. Cereb. Blood Flow Metab..

[38] Ando K., Oki E., Zhao Y., Ikawa-Yoshida A., Kitao H., Saeki H., Kimura Y., Ida S., Morita M., Kusumoto T. (2014). Mortalin is a prognostic factor of gastric cancer with normal p53 function. Gastric Cancer.

[39] Cheng W., Zhang B., Zikeliyar M., Wang J., Jian H., Wu K., Zhang Y., Ding J. (2019). Elevated Mortalin correlates with poor outcome in hepatocellular carcinoma. Ann. Diagn. Pathol..

[40] Taurin S., Seyrantepe V., Orlov S.N., Tremblay T.L., Thibault P., Bennett M.R., Hamet P., Pshezhetsky A.V. (2002). Proteome analysis and functional expression identify mortalin as an antiapoptotic gene induced by elevation of [Na+] i/[K+] i ratio in cultured vascular smooth muscle cells. Circ. Res..

[41] Zhang R., Meng Z., Wu X., Zhang M., Zhang S., Jin T. (2021). Mortalin promotes breast cancer malignancy. Exp. Mol. Pathol..

[42] Deocaris C., Lu W.J., C Kaul S., Wadhwa R. (2013). Druggability of mortalin for cancer and neuro-degenerative disorders. Curr. Pharm. Des..

[43] Elwakeel A. (2022). Abrogating the interaction between p53 and mortalin (Grp75/HSPA9/mtHsp70) for cancer therapy: The story so far. Front. Cell Dev. Biol..

[44] Dumont P., Leu J.L., Della Pietra A.C., George D.L., Murphy M. (2003). The codon 72 polymorphic variants of p53 have markedly different apoptotic potential. Nat. Genet..

[45] Mihara M., Erster S., Zaika A., Petrenko O., Chittenden T., Pancoska P., Moll U.M. (2003). p53 has a direct apoptogenic role at the mitochondria. Mol. Cell.

[46] Ichihara M., Murakumo Y., Takahashi M. (2004). RET and neuroendocrine tumors. Cancer Lett..

[47] Wu P.K., Hong S.K., Veeranki S., Karkhanis M., Starenki D., Plaza J.A., Park J.I. (2013). A mortalin/HSPA9-mediated switch in tumor-suppressive signaling of Raf/MEK/extracellular signal-regulated kinase. Mol. Cell Biol..

[48] Cano A., Pérez-Moreno M.A., Rodrigo I., Locascio A., Blanco M.J., del Barrio M.G., Portillo F., Nieto M.A. (2000). The transcription factor snail controls epithelial–mesenchymal transitions by repressing E-cadherin expression. Nat. Cell Biol..

[49] Mallini P., Lennard T., Kirby J., Meeson A. (2014). Epithelial-to-mesenchymal transition: What is the impact on breast cancer stem cells and drug resistance. Cancer Treat. Rev..

[50] Pizzatti L., Sá L.A., de Souza J.M., Bisch P.M., Abdelhay E. (2004). Altered protein profile in chronic myeloid leukemia chronic phase identified by a comparative proteomic study. Biochim. Biophys. Acta..

[51] Wang Y., Shi J., Chai K., Ying X., P Zhou B. (2013). The role of snail in EMT and tumorigenesis. Curr. Cancer Drug Targets.

[52] Na Y., Kaul S.C., Ryu J., Lee J.S., Ahn H.M., Kaul Z., Kalra R.S., Li L., Widodo N., Yun C.O. (2016). Stress chaperone mortalin contributes to epithelial-to-mesenchymal transition and cancer metastasis. Cancer Res..

[53] Rozenberg P., Kocsis J., Saar M., Prohászka Z., Füst G., Fishelson Z. (2013). Elevated levels of mitochondrial mortalin and cytosolic HSP70 in blood as risk factors in patients with colorectal cancer. Int. J. Cancer Res..

[54] Xu M., Zhang Y., Cui M., Wang X., Lin Z. (2020). Mortalin contributes to colorectal cancer by promoting proliferation and epithelial–mesenchymal transition. IUBMB Life.

[55] Yun C.O., Bhargava P., Na Y., Lee J.S., Ryu J., Kaul S.C., Wadhwa R. (2017). Relevance of mortalin to cancer cell stemness and cancer therapy. Sci. Rep..

[56] Rai R., Kennedy A.L., Isingizwe Z.R., Javadian P., Benbrook D.M. (2021). Similarities and Differences of Hsp70, hsc70, Grp78 and Mortalin as Cancer Biomarkers and Drug Target. Cells.

[57] Yoon A.R., Wadhwa R., Kaul S.C., Yun C.O. (2022). Why is mortalin a potential therapeutic target for cancer?. Front. Cell Dev. Biol..

[58] Chatterjee S., Burns T.F. (2017). Targeting heat shock proteins in cancer: A promising therapeutic approach. Int. J. Mol. Sci..

[59] Yang Y., Jin M., Dai Y., Shan W., Chen S., Cai R., Yang H., Tang L., Li L. (2021). Involvement and targeted intervention of mortalin-regulated proteome phosphorylated-modification in hepatocellular carcinoma. Front. Oncol..

[60] Kao T.Y., Chiu Y.C., Fang W.C., Cheng C.W., Kuo C.Y., Juan H.F., Wu S.H., Lee A.Y. (2015). Mitochondrial Lon regulates apoptosis through the association with Hsp60–mtHsp70 complex. Cell Death Dis..

[61] Karkhanis M., Park J.I. (2015). Sp1 regulates Raf/MEK/ERK-induced p21CIP1 transcription in TP53-mutated cancer cells. Cell. Signal..

[62] Teng M., Hu C., Yang B., Xiao W., Zhou Q., Li Y., Li Z. (2021). Salvianolic acid B targets mortalin and inhibits the migration and invasion of hepatocellular carcinoma via the RECK/STAT3 pathway. Cancer Cell Int..

[63] Sane S., Abdullah A., Nelson M.E., Wang H., Chauhan S.C., Newton S.S., Rezvani K. (2016). Structural studies of UBXN2A and mortalin interaction and the putative role of silenced UBXN2A in preventing response to chemotherapy. Cell Stress Chaperones.

[64] Sane S., Abdullah A., Boudreau D.A., Autenried R.K., Gupta B.K., Wang X., Wang H., Schlenker E.H., Zhang D., Telleria C. (2014). Ubiquitin-like (UBX)-domain-containing protein, UBXN2A, promotes cell death by interfering with the p53-Mortalin interactions in colon cancer cells. Cell Death Dis..

[65] Huang M.B., Brena D., Wu J.Y., Roth W.W., Owusu S., Bond V.C. (2022). Novel secretion modification region (SMR) peptide exhibits anti-metastatic properties in human breast cancer cells. Sci. Rep..

[66] Huang M.B., Wu J.Y., Lillard J., Bond V.C. (2019). SMR peptide antagonizes mortalin promoted release of extracellular vesicles and affects mortalin protection from complement-dependent cytotoxicity in breast cancer cells and leukemia cells. Oncotarget.

[67] Revel M., Daugan M.V., Sautés-Fridman C., Fridman W.H., Roumenina L.T. (2020). Complement system: Promoter or suppressor of cancer progression?. Antibodies.

[68] Mao X., Zhou L., Tey S.K., Ma A.P.Y., Yeung C.L.S., Ng T.H., Wong S.W.K., Liu B.H.M., Fung Y.M.E., Patz E.F. (2020). Tumour extracellular vesicle-derived Complement Factor H promotes tumorigenesis and metastasis by inhibiting complement-dependent cytotoxicity of tumour cells. J. Extracell. Vesicles.

[69] Hillman Y., Mazkereth N., Farberov L., Shomron N., Fishelson Z. (2016). Regulation of complement-dependent cytotoxicity by microRNAs miR-200b, miR-200c, and miR-217. J. Immunol..

[70] Fishelson Z., Kirschfink M. (2019). Complement C5b-9 and cancer: Mechanisms of cell damage, cancer counteractions, and approaches for intervention. Front. Immunol..

[71] Maruta H., Tikoo A., Shakri R., Shishido T. (1999). The anti-RAS cancer drug MKT-077 is an F-actin cross-linker. Ann. N. Y. Acad. Sci..

[72] Tikoo A., Cutler H., Lo S.H., Chen L.B., Maruta H. (1999). Treatment of Ras-induced cancers by the F-actin cappers tensin and chaetoglobosin K, in combination with the caspase-1 inhibitor N1445. Cancer J. Sci. Am..

[73] Chiba Y., Kubota T., Watanabe M., Otani Y., Teramoto T., Matsumoto Y., Koya K., Kitajima M. (1998). Selective antitumor activity of MKT-077, a delocalized lipophilic cation, on normal cells and cancer cells in vitro. J. Surg. Oncol..

[74] Moll U.M., Ostermeyer A.G., Haladay R., Winkfield B., Frazier M., Zambetti G. (1996). Cytoplasmic sequestration of wild-type p53 protein impairs the G1 checkpoint after DNA damage. Mol. Cell Biol..

[75] Ostermeyer A.G., Runko E., Winkfield B., Ahn B., Moll U.M. (1996). Cytoplasmically sequestered wild-type p53 protein in neuroblastoma is relocated to the nucleus by a C-terminal peptide. Proc. Natl. Acad. Sci. USA.

[76] Koya K., Li Y., Wang H., Ukai T., Tatsuta N., Kawakami M., Shishido T., Chen L.B. (1996). MKT-077, a novel rhodacyanine dye in clinical trials, exhibits anticarcinoma activity in preclinical studies based on selective mitochondrial accumulation. Cancer Res..

[77] Propper D.J., Braybrooke J.P., Taylor D.J., Lodi R., Styles P., Cramer J.A., Collins W.C.J., Levitt N.C., Talbot D.C., Ganesan T.S. (1999). Phase I trial of the selective mitochondrial toxin MKT 077 in chemo-resistant solid tumours. Ann. Oncol..

[78] Putri J.F., Bhargava P., Dhanjal J.K., Yaguchi T., Sundar D., Kaul S.C., Wadhwa R. (2019). Mortaparib, a novel dual inhibitor of mortalin and PARP1, is a potential drug candidate for ovarian and cervical cancers. J. Exp. Clin. Cancer Res..

[79] Aubrey B.J., Kelly G.L., Janic A., Herold M.J., Strasser A. (2018). How does p53 induce apoptosis and how does this relate to p53-mediated tumour suppression?. Cell Death Differ..

[80] Elwakeel A., Sari A.N., Dhanjal J.K., Meidinna H.N., Sundar D., Kaul S.C., Wadhwa R. (2021). Mutant p53(L194F) Harboring Luminal-A Breast Cancer Cells Are Refractory to Apoptosis and Cell Cycle Arrest in Response to Mortaparib(Plus), a Multimodal Small Molecule Inhibitor. Cancers.

[81] Sari A.N., Elwakeel A., Dhanjal J.K., Kumar V., Sundar D., Kaul S.C., Wadhwa R. (2021). Identification and characterization of mortaparib^Plus^—A novel triazole derivative that targets mortalin-p53 interaction and inhibits cancer-cell proliferation by wild-type p53-dependent and-independent mechanisms. Cancers.

[82] Kumar V., Sari A.N., Meidinna H.N., Dhanjal J.K., Subramani C., Basu B., Kaul S.C., Vrati S., Sundar D., Wadhwa R. (2021). Computational and in vitro experimental analyses of the Anti-COVID-19 potential of Mortaparib and Mortaparib^Plus^. Biosci. Rep..

[83] Meidinna H.N., Shefrin S., Sari A.N., Zhang H., Dhanjal J.K., Kaul S.C., Sundar D., Wadhwa R. (2022). Identification of a new member of Mortaparib class of inhibitors that target mortalin and PARP1. Front. Cell Dev. Biol..

[84] Benbrook D.M. (2022). SHetA2 attack on mortalin and colleagues in cancer therapy and prevention. Front. Cell Dev. Biol..

[85] Wadhwa R., Yang S., Meidinna H.N., Sari A.N., Bhargava P., Kaul S.C. (2024). Mixtures of Three Mortaparibs with Enhanced Anticancer, Anti-Migration, and Antistress Activities: Molecular Characterization in p53-Null Cancer Cells. Cancers.

[86] Park S.H., Baek K.H., Shin I., Shin I. (2018). Subcellular Hsp70 inhibitors promote cancer cell death via different mechanisms. Cell Chem. Biol..

[87] Smith R.A., Porteous C.M., Gane A.M., Murphy M.P. (2003). Delivery of bioactive molecules to mitochondria in vivo. Proc. Natl. Acad. Sci. USA.

[88] Benbrook D.M., Nammalwar B., Long A., Matsumoto H., Singh A., Bunce R.A., Berlin K.D. (2014). SHetA2 interference with mortalin binding to p66shc and p53 identified using drug-conjugated magnetic microspheres. Investig. New Drugs.

[89] Ko S.K., Kim S.K., Share A., Lynch V.M., Park J., Namkung W., Van Rossom W., Busschaert N., Gale P.A., Sessler J.L. (2014). Synthetic ion transporters can induce apoptosis by facilitating chloride anion transport into cells. Nat. Chem..

[90] Dewson G., Kluck R.M. (2009). Mechanisms by which Bak and Bax permeabilise mitochondria during apoptosis. J. Cell Sci..

[91] Moseng M.A., Nix J.C., Page R.C. (2019). 2-and N6-functionalized adenosine-5’-diphosphate analogs for the inhibition of Mortalin. FEBS Lett..

[92] Liu S., Zhou G., Lo S.N.H., Louie M., Rajagopalan V. (2016). SHetA2, a new cancer-preventive drug candidate. Anti-cancer Drugs: Nature, Synthesis and Cell. InTech.

[93] Nammalwar B., Berlin K.D., Bunce R.A. (2013). SHetA2—A mini review of a promising anticancer drug. JSM Chem..

[94] Benbrook D.M. (2002). Refining retinoids with heteroatoms. Mini-Rev. Med. Chem..

[95] Chun K.H., Benbrook D.M., Berlin K.D., Hong W.K., Lotan R. (2003). The synthetic heteroarotinoid SHetA2 induces apoptosis in squamous carcinoma cells through a receptor-independent and mitochondria-dependent pathway. Cancer Res..

[96] Guruswamy S., Lightfoot S., Gold M.A., Hassan R., Berlin K.D., Ivey R.T., Benbrook D.M. (2001). Effects of retinoids on cancerous phenotype and apoptosis in organotypic cultures of ovarian carcinoma. J. Natl. Cancer Inst..

[97] Liu S., Brown C.W., Berlin K.D., Dhar A., Guruswamy S., Brown D., Gardner G.J., Birrer M.J., Benbrook D.M. (2004). Synthesis of flexible sulfur-containing heteroarotinoids that induce apoptosis and reactive oxygen species with discrimination between malignant and benign cells. J. Med. Chem..

[98] Liu T., Hannafon B., Gill L., Kelly W., Benbrook D. (2007). Flex-Hets differentially induce apoptosis in cancer over normal cells by directly targeting mitochondria. Mol. Cancer Ther..

[99] Liu T., Masamha C.P., Chengedza S., Berlin K.D., Lightfoot S., He F., Benbrook D.M. (2009). Development of flexible-heteroarotinoids for kidney cancer. Mol. Cancer Ther..

[100] Lin Y., Liu X., Yue P., Benbrook D.M., Berlin K.D., Khuri F.R., Sun S.Y. (2008). Involvement of c-FLIP and survivin down-regulation in flexible heteroarotinoid-induced apoptosis and enhancement of TRAIL-initiated apoptosis in lung cancer cells. Mol. Cancer Ther..

[101] Orsini F., Migliaccio E., Moroni M., Contursi C., Raker V.A., Piccini D., Martin-Padura I., Pelliccia G., Trinei M., Bono M. (2004). The life span determinant p66Shc localizes to mitochondria where it associates with mitochondrial heat shock protein 70 and regulates trans-membrane potential. J. Biol. Chem..

[102] Zhang H., Tang Y., Zhang Y., Zhang S., Qu J., Wang X., Kong R., Han C., Liu Z. (2015). Fucoxanthin: A promising medicinal and nutritional ingredient. J. Evid. Based Complement. Altern..

[103] Wang L., Zeng Y., Liu Y., Hu X., Li S., Wang Y., Li L., Lei Z., Zhang Z. (2014). Fucoxanthin induces growth arrest and apoptosis in human bladder cancer T24 cells by up-regulation of p21 and down-regulation of mortalin. Acta Biochim. Biophys. Sin..

[104] Abdullah A., Sane S., Branick K.A., Freeling J.L., Wang H., Zhang D., Rezvani K. (2015). A plant alkaloid, veratridine, potentiates cancer chemosensitivity by UBXN2A-dependent inhibition of an oncoprotein, mortalin-2. Oncotarget.

[105] Narayanaswamy R., Shymatak M., Chatterjee S., Wai L.K., Arumugam G. (2014). Inhibition of Angiogenesis and nitric oxide synthase (NOS), by embelin & vilangin using in vitro, in vivo & in silico studies. Adv. Pharm. Bull..

[106] Tang Q., Zhong H., Xie F., Xie J., Chen H., Yao G. (2014). Expression of miR-106b-25 induced by salvianolic acid B inhibits epithelial-to-mesenchymal transition in HK-2 cells. Eur. J. Pharmacol..

[107] Kaul S.C., Wadhwa R. (2017). Science of Ashwagandha: Preventive and Therapeutic Potentials.

[108] Sari A.N., Dhanjal J.K., Elwakeel A., Kumar V., Meidinna H.N., Zhang H., Ishida Y., Terao K., Sundar D., Kaul S.C. (2022). A low dose combination of withaferin A and caffeic acid phenethyl ester possesses anti-metastatic potential in vitro: Molecular targets and mechanisms. Cancers.

[109] Chen J.C., Chiu M.H., Nie R.L., Cordell G.A., Qiu S.X. (2005). Cucurbitacins and cucurbitane glycosides: Structures and biological activities. Nat. Prod. Rep..

[110] Pham M.Q., Tran T.H.V., Pham Q.L., Gairin J.E. (2019). In silico analysis of the binding properties of solasonine to mortalin and p53, and in vitro pharmacological studies of its apoptotic and cytotoxic effects on human HepG2 and Hep3b hepatocellular carcinoma cells. Fundam. Clin. Pharmacol..

[111] Ahmed Hamdi O.A., Syed Abdul Rahman S.N., Awang K., Abdul Wahab N., Looi C.Y., Thomas N.F., Abd Malek S.N. (2014). Cytotoxic constituents from the rhizomes of Curcuma zedoaria. Sci. World J..

[112] Fitriana N., Khairunnisa F.A., Rifa’i M., Widodo (2019). Potential compound of Curcuma xanthorrhiza and Curcuma zedoaria as Mortalin inhibitor to control cancer cell growth through computational study. IOP Conf. Ser. Earth Environ. Sci..

[113] Hartati F.K., Djauhari A.B. (2020). Potential of black rice (*Oryza sativa* L.) as anticancer through mortalin-p53 complex inhibitors. Biointerface Res. Appl. Chem.

[114] Nagpal N., Goyal S., Dhanjal J.K., Ye L., Kaul S.C., Wadhwa R., Chaturvedi R., Grover A. (2017). Molecular dynamics-based identification of novel natural mortalin–p53 abrogators as anticancer agents. J. Recept. Signal Transduct..

[115] Wen W., Liu W., Shao Y., Chen L. (2014). VER-155008, a small molecule inhibitor of HSP70 with potent anti-cancer activity on lung cancer cell lines. Exp. Biol. Med..

[116] Tang X., Tan L., Shi K., Peng J., Xiao Y., Li W., Chen L., Yang Q., Qian Z. (2018). Gold nanorods together with HSP inhibitor-VER-155008 micelles for colon cancer mild-temperature photothermal therapy. Acta Pharm. Sin. B.

[117] Fewell S.W., Day B.W., Brodsky J.L. (2001). Identification of an inhibitor of hsc70-mediated protein translocation and ATP hydrolysis. J. Biol. Chem..

[118] Prince T., Ackerman A., Cavanaugh A., Schreiter B., Juengst B., Andolino C., Danella J., Chernin M., Williams H. (2018). Dual targeting of HSP70 does not induce the heat shock response and synergistically reduces cell viability in muscle invasive bladder cancer. Oncotarget.

[119] Huryn D.M., Brodsky J.L., Brummond K.M., Chambers P.G., Eyer B., Ireland A.W., Kawasumi M., LaPorte M.G., Lloyd K., Manteau B. (2011). Chemical methodology as a source of small-molecule checkpoint inhibitors and heat shock protein 70 (Hsp70) modulators. Proc. Natl. Acad. Sci. USA.

[120] Goloudina A.R., Demidov O.N., Garrido C. (2012). Inhibition of HSP70: A challenging anti-cancer strategy. Cancer Lett..

[121] Leu J.J., Pimkina J., Frank A., Murphy M.E., George D.L. (2009). A small molecule inhibitor of inducible heat shock protein 70. Mol. Cell.

[122] Leu J.I.J., Pimkina J., Pandey P., Murphy M.E., George D.L. (2011). HSP70 inhibition by the small-molecule 2-phenylethynesulfonamide impairs protein clearance pathways in tumor cells. Mol. Cancer Res..

[123] Colvin T.A., Gabai V.L., Gong J., Calderwood S.K., Li H., Gummuluru S., Matchuk O.N., Smirnova S.G., Orlova N.V., Zamulaeva I.A. (2014). Hsp70–Bag3 interactions regulate cancer-related signaling networks. Cancer Res..

[124] Koren J., Miyata Y., Kiray J., O’Leary J.C., Nguyen L., Guo J., Blair L.J., Li X., Jinwal U.K., Cheng J.Q. (2012). Rhodacyanine derivative selectively targets cancer cells and overcomes tamoxifen resistance. PLoS ONE.

[125] Li X., Srinivasan S.R., Connarn J., Ahmad A., Young Z.T., Kabza A.M., Zuiderweg E.R., Sun D., Gestwicki J.E. (2013). Analogues of the allosteric heat shock protein 70 (Hsp70) inhibitor, MKT-077, as anti-cancer agents. ACS Med. Chem. Lett..

[126] Yaglom J.A., Wang Y., Li A., Li Z., Monti S., Alexandrov I., Lu X., Sherman M.Y. (2018). Cancer cell responses to Hsp70 inhibitor JG-98: Comparison with Hsp90 inhibitors and finding synergistic drug combinations. Sci. Rep..

[127] Oz M., Lorke D.E., Hasan M., Petroianu G.A. (2011). Cellular and molecular actions of methylene blue in the nervous system. Med. Res. Rev..

[128] Wang A.M., Morishima Y., Clapp K.M., Peng H.M., Pratt W.B., Gestwicki J.E., Osawa Y., Lieberman A.P. (2010). Inhibition of hsp70 by methylene blue affects signaling protein function and ubiquitination and modulates polyglutamine protein degradation. J. Biol. Chem..

[129] Thomas S., Sharma N., Gonzalez R., Pao P.W., Hofman F.M., Chen T.C., Louie S.G., Pirrung M.C., Schönthal A.H. (2013). Repositioning of verrucosidin, a purported inhibitor of chaperone protein GRP78, as an inhibitor of mitochondrial electron transport chain complex I. PLoS ONE.

[130] Martin S., Lamb H.K., Brady C., Lefkove B., Bonner M.Y., Thompson P., Lovat P.E., Arbiser J.L., Hawkins A.R., Redfern C.P.F. (2013). Inducing apoptosis of cancer cells using small-molecule plant compounds that bind to GRP78. Br. J. Cancer.

[131] Yuan Z.P., Chen L.J., Fan L.Y., Tang M.H., Yang G.L., Yang H.S., Du X.B., Wang G.Q., Yao W.X., Zhao Q.M. (2006). Liposomal quercetin efficiently suppresses growth of solid tumors in murine models. Clin. Cancer Res..

[132] Choi D.W., Lim M.S., Lee J.W., Chun W., Lee S.H., Nam Y.H., Park J.M., Choi D.H., Kang C.D., Lee S.J. (2015). The cytotoxicity of kahweol in HT-29 human colorectal cancer cells is mediated by apoptosis and suppression of heat shock protein 70 expression. Biomol. Ther..

[133] Kim J.A., Kim Y., Kwon B.M., Han D.C. (2013). The natural compound cantharidin induces cancer cell death through inhibition of heat shock protein 70 (HSP70) and Bcl-2-associated athanogene domain 3 (BAG3) expression by blocking heat shock factor 1 (HSF1) binding to promoters. J. Biol. Chem..

[134] Fasano E., Serini S., Piccioni E., Toesca A., Monego G., Cittadini A.R., Ranelletti F.O., Calviello G. (2012). DHA induces apoptosis by altering the expression and cellular location of GRP78 in colon cancer cell lines. Biochim. Biophys. Acta Mol. Basis Dis..

[135] Chang Y., Sui J., Fu Q., Lu Z., Piao Z., Jin T., Zhang M. (2024). Mortalin promotes the evolution of androgen-independent prostate cancer through Wnt/beta-catenin signaling pathway. Cancer Cell Int..

[136] Shankaranarayana A.H., Meduri B., Pujar G.V., Hariharapura R.C., Sethu A.K., Singh M., Bidye D. (2023). Restoration of p53 functions by suppression of mortalin-p53 sequestration: An emerging target in cancer therapy. Future Med. Chem..

[137] Avraham M., Sinkovits G., Hurler L., Prohászka Z., Fishelson Z. (2024). Circulating mortalin in blood and activation of the alternative complement pathway as risk indicators in COVID-19 infection. Front. Immunol..

[138] Londono C., Osorio C., Gama V., Alzate O. (2012). Mortalin, apoptosis, and neurodegeneration. Biomolecules.

[139] Flachbartová Z., Kovacech B. (2013). Mortalin—A multipotent chaperone regulating cellular processes ranging from viral infection to neurodegeneration. Acta Virol..

[140] Priyanka, Seth P. (2022). Insights into the Role of Mortalin in Alzheimer’s Disease, Parkinson’s Disease, and HIV-1-Associated Neurocognitive Disorders. Front. Cell Dev. Biol..

